# Environmental evidence in action: on the science and practice of evidence synthesis and evidence-based decision-making

**DOI:** 10.1186/s13750-023-00302-5

**Published:** 2023-05-18

**Authors:** Steven J. Cooke, Carly N. Cook, Vivian M. Nguyen, Jessica C. Walsh, Nathan Young, Christopher Cvitanovic, Matthew J. Grainger, Nicola P. Randall, Matt Muir, Andrew N. Kadykalo, Kathryn A. Monk, Andrew S. Pullin

**Affiliations:** 1 Canadian Centre for Evidence-Based Conservation, Department of Biology and Institute of Environmental and Interdisciplinary Science, Carleton University, 1125 Colonel By Dr., Ottawa, ON K1S 5B6 Canada; 2 School of Biological Sciences, Monash University, 25 Rainforest Walk, Clayton, VIC 3800 Australia; 3 School of Sociological and Anthropological Studies, University of Ottawa, 20 University Private, Ottawa, ON K1N 6N5 Canada; 4 School of Business, University of New South Wales, Canberra, ACT 2612 Australia; 5 Norwegian Institute for Nature Research-NINA, Torgarden, 5685, 7485 Trondheim, Norway; 6 Centre for Evidence Based Agriculture, Harper Adams University, Newport, Shropshire, TF10 8NB UK; 7 U.S. Fish & Wildlife Service, 5275 Leesburg Pike, Falls Church, VA 22041-3803 USA; 8 Department of Natural Resource Sciences, McGill University, 111 Lakeshore Blvd, Ste-Anne-de-Bellevue, QC H9X 3V9 Canada; 9 Department of Biosciences, Swansea University, Swansea, SA2 8PP UK; 10 School of Natural Sciences, Bangor University, Bangor, LL57 2DG UK

**Keywords:** Implementation science, Evidence, Practice, Policy, Synthesis

## Abstract

In civil society we expect that policy and management decisions will be made using the best available evidence. Yet, it is widely known that there are many barriers that limit the extent to which that occurs. One way to overcome these barriers is via robust, comprehensive, transparent and repeatable evidence syntheses (such as systematic reviews) that attempt to minimize various forms of bias to present a summary of existing knowledge for decision-making purposes. Relative to other disciplines (e.g., health care, education), such evidence-based decision-making remains relatively nascent for environment management despite major threats to humanity, such as the climate, pollution and biodiversity crises demonstrating that human well-being is inextricably linked to the biophysical environment. Fortunately, there are a growing number of environmental evidence syntheses being produced that can be used by decision makers. It is therefore an opportune time to reflect on the science and practice of evidence-based decision-making in environment management to understand the extent to which evidence syntheses are embraced and applied in practice. Here we outline a number of key questions related to the use of environmental evidence that need to be explored in an effort to enhance evidence-based decision-making. There is an urgent need for research involving methods from social science, behavioural sciences, and public policy to understand the basis for patterns and trends in environmental evidence use (or misuse or ignorance). There is also a need for those who commission and produce evidence syntheses, as well as the end users of these syntheses to reflect on their experiences and share them with the broader evidence-based practice community to identify needs and opportunities for advancing the entire process of evidence-based practice. It is our hope that the ideas shared here will serve as a roadmap for additional scholarship that will collectively enhance evidence-based decision-making and ultimately benefit the environment and humanity.

## Introduction

On the surface, the concept of evidence-based decision-making is simple—evidence is used to help make decisions. However, the reality is much more complex. Evidence comes in many forms and it is easy to intentionally make selective use of evidence to support a position. For that reason, evidence-based decision-making should be based on the synthesis of all available evidence. However, not all evidence syntheses are robust and can themselves introduce bias [1]. Moreover, individual empirical studies vary in validity [2]. The reproducibility crisis in science (see [3]) reinforces that just because a paper is published does not mean its conclusion is valid. In many cases experimental designs can limit inferences and the conclusions that authors can draw from their work. Or in other words, science is imperfect and so is the peer review process which is tasked with upholding high standards for robust evidence [4]. Fortunately, there are now robust, transparent and repeatable methods that exist for conducting comprehensive evidence syntheses (i.e., systematic reviews; although other forms of evidence synthesis also have merit depending on their use) that reduce risk of bias. Such syntheses have revolutionized health care and are applicable to a wide range of disciplines, including those focused on environmental issues. However, there are also other forms of evidence synthesis that involve various short cuts (e.g. rapid reviews) and with them comes greater potential for bias and uncertainty [5]. There is no shortage of environmental challenges that require robust policy and management decisions to ensure interventions are effective and do not squander limited resources.

As the environmental sector begins to embrace the notion of evidence-based decision-making [6, 7], it is important to understand and embrace various processes that underpin evidence generation, synthesis and use. It is therefore an opportune time to reflect on the science and practice of evidence-based decision-making in environment management to understand the extent to which it is embraced and applied in practice. Here we outline 13 key questions related to the use of environmental evidence that need to be addressed in an effort to enhance evidence-based decision-making in the environmental sphere. Given the importance of evidence synthesis as a pillar of evidence-based decision-making [8], these topics are discussed in an integrated manner.

## Key questions on the science and practice of evidence-based decision-making related to the environment

We present each key question along with a brief overview of the context for the questions and potential approaches for answering them. Who addresses these questions varies with each question. In some cases it is the “researcher” while in others it is the broader evidence synthesis community. This is a compilation of ideas based on the lived experiences, knowledge, and perspectives of the international team of authors. All of the authors work in some aspect of the evidence generation, synthesis and application ecosystem.

### How should different types of evidence be weighted, judged, or considered differently in evidence synthesis and decision-making?

There are many different forms of evidence, including scientific, expert, experiential, local and Indigenous knowledge [9, 10]. Each of these knowledges are critical inputs into environmental decisions. All of these types of evidence can provide environmental, social, economic and practical information relevant to a decision, and can be further categorised based on their source and validity. For example, with respect to validity, scientific evidence can be found in published or unpublished primary studies, reviews, summaries, decision-support tools or guidelines [11, 12]. Several factors determine the validity of evidence, including study/review design, sample size, methods to reduce biases, and external validity [13, 14]. While the importance of critically appraising the validity of evidence is well known in the evidence synthesis community [15], it is unclear whether practitioners and policy makers in the environmental sphere take this into account. Understanding how different actors engage with environmental evidence is also a valid question. A key knowledge gap in environmental decision-making is an understanding of how influential evidence type, source and validity are in deciding which evidence to use, and how much weight decision makers assign to each factor (if at all). Relatedly, understanding which forms of evidence are most appropriate for different contexts (and with different actors) also deserves more consideration (i.e., What is fit-for-purpose for environmental decision-making?).

### What are the solutions to established barriers limiting evidence-based decision-making and how do they vary in different contexts?

Contrary to what many in the scientific research community might assume, evidence, especially peer-reviewed science, is rarely the first or most widely used nor the most valued source of information considered in environmental decisions [16]. Several recent studies have used surveys or interviews with environmental managers to understand factors that facilitate or limit the use of environmental evidence. The most common barriers to environmental evidence use in decision-making are accessibility of the evidence; relevance and applicability of the evidence; organizational capacity, resources, and finances; time required to find and read evidence; and poor communication and dissemination skills between scientists and decision makers [16, 17]. These barriers are inventoried in a comprehensive ‘typology of barriers and enablers of evidence use’ [18]. Several practical solutions have been proposed to overcome ‘evidence complacency’ (defined as “a way of working in which, despite availability, evidence is not sought or used to make decisions, and the impact of actions is not tested” [19]). However, little is known about which of these potential solutions work most effectively for environmental management. New tools like Evidence-to-Decision [E2D] tool (www.evidence2decisiontool.com) have been developed to guide practitioners through a structured process to transparently document and report the evidence that contributes to decisions (see [20]). Future research is needed on which solutions effectively transform barriers to evidence-informed decision-making into enablers, and specifically, how each of these enablers facilitate the use of scientific evidence in practice [18]. Determining how potential evidence users procure information given capacity shortages and information overload is important. A study on the use of Conservation Evidence’s (https://www.conservationevidence.com/) subject-wide evidence syntheses found that well-summarized evidence can direct management choices away from ineffective interventions when it is timely and packaged in a form that meets the needs of practitioners [21]. The same approach could be adopted, for example, to investigate whether brokered or co-assessed or co-produced evidence influences practitioner decision-making and the role of evidence in decisions.

### How are evidence syntheses viewed by different users and user groups? And what determines whether such syntheses will be embraced and used?

At present, it is unclear how evidence syntheses are viewed by different users and user groups. This is troublesome in that without such knowledge it is difficult to know why syntheses may be embraced or ignored. We acknowledge here that such questions are context specific and will undoubtedly vary whether being asked in the global south or global north. Of course, there are various types/methods for evidence synthesis, and each comes with caveats. For example, to what extent are users familiar with the caveats that come with, for example rapid versus comprehensive systematic reviews? Thomas-Walters et al. [22] revealed that Canadian environmental policy makers were generally familiar with the suite of evidence synthesis tools available and would embrace systematic reviews over other methods if they were available for a given topic and in a reasonable timeframe. It is unclear how widespread that perspective is (e.g., in other jurisdictions) and whether it extends to front-line practitioners, although a commentary paper authored primarily by UK government scientific advisors suggested four principles to enhance the use and value of evidence syntheses for policymakers. The suggested principles are to be inclusive, transparent, rigorous, and accessible [23]. Collins et al. [24] also investigated the perception of UK policy makers to evidence syntheses and suggested that co-production between the review experts and policy teams facilitate both better creation of evidence syntheses, and better use of the final product. Collins et al. [24] also suggested that policy relevant reviews may require the trade-offs between rigour and timeliness, but that this could be managed through risk-based approaches to the methodology. Communicating uncertainty that arises from different methodological choices will most certainly require nuance. What is the best way to do so in ways that do not unnecessarily discredit fit-for-purpose tools yet also do not create a standard whereby biased forms of evidence syntheses become the norm in decision-making? Specific efforts to contrast and compare efforts in the global south and north would be useful for understanding the generality of potential solutions for enhancing uptake of evidence syntheses.

### Have evidence syntheses influenced environmental policy and decision? If so—how? And if not—What could be done to make evidence syntheses more useful to policy makers and environmental managers?

The assumption is that evidence syntheses are used by environmental policy managers and decision makers [25]. This is especially the case if they have commissioned a synthesis. Yet, research has revealed that although they understand and value high quality evidence syntheses, rarely are they available for a topic of interest when they are needed and there can be institutional barriers to integrating evidence synthesis into existing decision-making frameworks [22]. The health care realm has processes for integrating the outcomes of syntheses into standardized best practice (clinical) guidelines [26] yet similar processes are lacking for the environmental sphere. When the authors of systematic reviews dealing with environmental topics were queried about if and how their work influenced policy and practice, about half of respondents were able to identify tangible application [27]. Even if there is a systematic review available for a topic of relevance to management, there can be a variety of barriers that limit use related to lack of capacity for uptake. When decision makers are involved in shaping the review question and scope, and are engaged throughout the systematic review process (recognizing that there can be a fine line between engagement and interference—for example, biasing outcomes), there seems to be a greater likelihood of syntheses being embraced (i.e., used to inform decisions [27]). Efforts to understand and improve translation of evidence syntheses to knowledge users for their specific context would be beneficial. The outcomes from evidence syntheses are often “it depends”, so there is need for better messaging and exploring ways in which evidence synthesis outputs can be shared, perhaps in visual formats. In some cases, evidence synthesis can provide general guidance whereas in other cases the nuance around “when does it work” is crucial. How we communicate these different outcomes is highly relevant to communicating uncertainty to various audiences. Given the importance of this question(s), there is need for much research around fully understanding the barriers and enablers to use of evidence syntheses (as per [18]) as well as the development of measures and indicators for assessing impact of evidence syntheses over time so as to elevate the impact of future evidence syntheses resulting in better environmental policy and decisions. Similarly, it would be useful to have objective indicators of evidence use along with better mechanisms to evaluate impact and success. That alone represents a major research need. Understanding not only if evidence is used but also how it is used is critical to overcoming barriers.

### What can be done to build public trust in evidence and evidence syntheses so that decision makers are empowered to embrace them?

Public trust in political and scientific authorities has declined significantly over the past several decades across most democratic societies [28]. Causes are complex, including resurgent populism, political polarization, culture war struggles, and the expansion of anti-establishment alternatives and social media [29]. Pushing back against these trends to build public trust and acceptance of evidence and evidence syntheses will be challenging. When it comes to evidence synthesis, presumably public understanding is even less clear emphasizing need for building understanding. During the COVID-19 pandemic the concept of evidence synthesis was normalized (see [30]) such that there may be opportunity to leverage that understanding in an environmental context. Existing research suggests that transparency is critical [31]. This includes transparency of sponsorship (who has asked for evidence and for what purpose), transparency of process (how syntheses are performed and by whom), and transparency of outcome (to allow evaluations of the reliability and validity of syntheses). Biographical profiles of the researchers should be publicly available, as well as all methods, codebooks and data summaries. Whenever possible, lay language summaries should be produced as part of the synthesis process. Lay language text should be traceable (using hyperlinks for example) to detailed scientific descriptions and original sources. Transparency is a necessary but not sufficient criterion for building public trust. Public controversy or mistrust are more likely when the stakes are higher and decisions may lead to losses of income or opportunity, as is often the case with environmental governance [32]. Political leaders and bureaucratic decision makers must offer rhetorical support for evidence synthesis while also emphasizing that these are important tools for decision-making and not substitutes for political processes such as stakeholder and rights-holder consultations.

### What aspects of environmental evidence syntheses are considered particularly important and useful by evidence users?

There are many aspects of the evidence synthesis process that might make them more or less valued by evidence users. The emphasis of systematic review methodology is on estimating the truth whilst reducing risk of bias through replicability of methods, transparency of reporting and critical appraisal of study validity. The extent to which this approach results in useful information to the users depends primarily on the extent and validity of the existing primary evidence. Other aspects such as relevance to need, cost, timeliness, accessibility, certainty, might be of higher priority to an evidence user. Trade-offs in the reliability of evidence and these other characteristics will differ depending on the pressures of the decision-making process (e.g. the risk of making the wrong decision). There is a gap in dialogue between producers and users on how to prioritise these aspects when planning syntheses. Co-production of evidence syntheses is often promoted as a way of selecting both a relevant and valid question and the appropriate synthesis method (e.g. rapid versus systematic review or Mapping evidence versus estimating effects) is a logical next step. Investment in general services (e.g. CEEDER; https://environmentalevidence.org/ceeder/) or in living evidence syntheses’ (see below) is unlikely to be attractive to individual organisations and may require consortia to ensure aspects of low cost, timeliness and accessibility are provided. Nonetheless, such efforts should be pursued.

### How can outputs from evidence syntheses best be shared or translated for different audiences in ways that ensure they remain current and accessible?

Evidence syntheses provide a vital role in decision-making by summarising a body of evidence linked to a decision-context. As such they often need to communicate complex outcomes (including uncertainty) to a wide range of interested parties, which could include policymakers, local decision makers (e.g. Nature reserve managers), local people and the public at larger. One effective form of communication is through the use of diagrams, as they help the user understand the evidence synthesis question(s) and results [33]. Increasingly, evidence synthesists take advantage of tools to create interactive diagrams. Interactivity facilitates learning and understanding [34], making evidence synthesis more accessible to decision makers. Tools such as EviAtlas [35], an OpenSource tool for visualising databases of systematic evidence syntheses, allow a low technical barrier to reviewers to create high quality, shareable data visualizations. In some cases the speed at which evidence syntheses can be produced is out-paced by the speed at which new primary evidence is produced. Syntheses can even be out of date as soon as they are published. For example, 7% of 100 systematic reviews assessed in Shojania et al. [36] were out of date at the time of publication. In these cases, “living reviews” have been suggested as a potential solution [37]. A “living review” is an online publicly available high quality systematic review which is updated as new evidence becomes available. In order to produce a living review there needs to be consideration of the workflow (teams need to be responsive to new evidence), and analysis methods (to avoid potential high false-positive rates and unstable effect size estimates). It is important to note that in order for living reviews to be truly effective in environmental fields there needs to be a greater emphasis on standardized reporting in the primary literature (e.g., effect sizes—so that publications are more machine readable; [38].

### How can boundary-spanning support the translation and uptake of evidence syntheses among different audiences?

Boundary spanning is one of many approaches intended to enable evidence-based decision-making. It is a concept that emerged from the business and organizational management fields in the 1970s to facilitate knowledge sharing and exchange between two or more entities [39]. In the field of environmental research and management, boundary spanning has been defined as ‘work to enable exchange between the production and use of knowledge to support evidence-informed decision-making in a specific context’ [40]. Further, there exist entities, individuals or organizations that may work specifically to facilitate this process called boundary spanning (relationship-building entities) that may operate along side of knowledge brokers (engaged in multiple functions) or intermediaries (research-disseminating [41]). Boundary spanners have recently been acknowledged as key players in making knowledge more actionable in the environmental realm [42] yet more work is needed to better elucidate their role in evidence-based decision-making [43]. Furthermore, there exist boundary objects which are artifacts that cross boundaries to fulfil a bridging function [44]. Tools, strategies or frameworks developed that support the bridging between two boundaries (e.g., evidence producers vs users) may be considered boundary objects and may play an important role in promoting uptake of evidence. Thus, questions we ask must include boundary spanning actors, objects and even processes. Examples of outstanding questions include: What are the key attributes of boundary spanners? What boundary objects are used by different audiences and how do they support evidence uptake? Answering these questions would help to determine the best mechanisms for use of boundary-spanning to achieve evidence-based environmental management.

### What can be done to create a culture of evidence use and evidence-based decisions among individuals and organizations?

It is one thing to provide evidence users with evidence in reliable and usable forms (such as systematic reviews), yet underpinning that is the need to create a culture of evidence use among individuals and organizations such that robust syntheses are embraced and used. The outstanding question—is how to do so? Little is known about this in the context of environmental evidence emphasizing much scope for empirical research with key informants. However, there are learnings and lessons from other knowledge domains. In mental health, clinicians are required by insurers to provide evidence that interventions being used are effective—a “top down” mechanism for creating (or forcing) a culture of evidence use and assessment of effectiveness [45]. The creation of feedback systems required clinicians to work closely with researchers which further increased knowledge exchange and better understanding of evidence needs [45]. In healthcare management, a multi-faceted approach to creating a culture of evidence use benefitted from leadership, funding, infrastructure (creating spaces for collaboration), staff development, partnerships, and change management [46]. In nursing, the enculturalization of evidence-based practice involved providing team members with the resources and support needed to do so [47]. Some domains have suggested greater education, mentorship, and skill-building related to evidence use among actors to support a culture of evidence use. For example, including evidence-based practice content in study programs and professional education, designing curricula to foster attributes known to support evidence-based practice and decisions [48]. Understanding what individual characteristics and aspects of organizational structures and training create a culture of evidence use and evidence-based decision-making is necessary to foster and enable such enculturement to occur. Reflective research focused on instances where this has (or has not) occurred combined with experimental interventions with relevant comparators (e.g., before and after) will help to inform the development of a culture of evidence use and evidence-based decisions in the environmental sector.

### What type of training would best prepare environmental decision makers for embracing evidence-based decision-making?

Decision makers are increasingly aware of the need to make their work evidence informed but, with a suite of different methods and terminologies for synthesis methods, it can be difficult for potential users of evidence to know which methods to select to inform decisions, and how reliable or appropriate each may be. There are tools and resources that can help support decision makers in this. For example, the EU Eklipse Mechanism has created a summary of different knowledge synthesis methods, together with their respective costs, time requirements and robustness [49], but formal training may also be valuable for users of specific evidence collation and synthesis methods. Recent developments specific to the environmental sector include a call for better training in evidence synthesis and evidence-based management [25, 50, 51] as well as a summary of key learning objectives and associated resources to help with delivery ([51]; https://www.britishecologicalsociety.org/applied-ecology-resources/about-aer/additional-resources/evidence-in-conservation-teaching/). Training specific to evidence-based medicine has been sufficiently well studied that Illic and Maloney [52] were able to conduct a systematic review on the topic. They revealed that learner competency in evidence-based medicine increased following various interventions (e.g., in person lecture, online, self-directed, group learning, etc.), yet there was no particular mode of delivery that performed better than others. We are a long way from being able to do a similar systematic review in the context of environmental evidence but there is much room to grow the evidence base. Relatedly, there is need to identify the specific skills and attributes that need to be fostered and developed in training sessions to ensure that training is effective.

### What government policies promote evidence-based decision-making within government?

How government policymakers use evidence in their decision-making has long been the focus of scholarship [53]. Yet, may questions remain about what is happening within government to identify the policies, laws and funding mechanisms that promote the practices and improve the quality of evidence-based decision-making. For example, the Federal legislative branch in the United States passed the 2018 law, The Foundations for Evidence-Based Policymaking Act (abbreviated as the Evidence Act), which was followed by executive guidance and orders to promote “evidence-based decisions guided by the best available science and data” [54] and to direct Federal agencies to identify and set priorities for evidence building [55, 56]. A broader set of government policies may be relevant as evidence-adjacent. For example, should government initiatives that foster Open Data, scientific integrity, and performance measurement be considered as promoting evidence-based decision-making within government? Finally, what is the role of a government’s budgeting process: what funding mechanisms and practices (e.g., performance-based budgeting) promote evidence-based decision-making within government? These questions are germane to decision-making and regulatory bodies within national, provincial and state governments, as well as decision-making related to the implementation of multilateral treaties and conventions.

### How do we weave different types of knowledge in or alongside evidence syntheses?

So called “modern scientific knowledge” predominates in evidence syntheses for good reason. Such knowledge is often shared via peer reviewed publications that are archived, searchable, and accessible. Ideally such research is conducted in a manner that minimizes biases. Moreover, there are accepted protocols for evaluating and synthesizing such evidence. Yet, other forms of knowledge such as Indigenous science and wisdom and local knowledge are also valid and valued forms of evidence. So how do we weave or braid different types of knowledge (beyond western science) in evidence syntheses? There are a growing number of paradigms (e.g., two-eyed seeing [57]; that attempt to weave knowledge systems in respectful ways [58]). Although admirable, such efforts have yet to be fully refined to work in the context of evidence-based decision-making where there is a reliance on actions such as critical appraisal that assess study validity (e.g., is there a relevant comparator, is there replication). Assessing “validity” and reliability of Indigenous and local knowledge is entirely different. A series of recent systematic maps and reviews have explored knowledge bridging (e.g. [59–61]), yet none have fully considered how such knowledge can be woven into systematic reviews alongside western science. For example, is the best approach to involve Indigenous knowledge holders in question setting and interpretation or to attempt to have Indigenous and western science formally included as sources of evidence within a synthesis? Other sources have begun to consider how to incorporate different types of knowledge or evidence in environmental decision-making. The checklists provided by the EU Eklipse Mechanism [49] are designed to help decision makers determine which method(s) of knowledge and/or evidence collation might help support different types of questions/decisions, and questions around what counts as evidence and how to weight different types of evidence are beginning to be considered by others (see [7]). Much more work is needed in this space including scholarship around how to assess and value different knowledge sources in evidence syntheses in ways that are respectful and rigorous. As with many of the questions covered here, there has been more work in the health domain [62] but we are beginning to reconcile with these questions in the environmental sphere [63]. Failure to do so will alienate knowledge holders, impede Indigenous knowledge sovereignty and impede our ability to achieve inclusive evidence-based decision-making [64].

### What are the ethical considerations for environmental evidence syntheses?

Evidence syntheses play an increasingly important role in consolidating and disseminating research knowledge with a view to informing policy, practice and public perception. Significant efforts have been made to enhance the methodological rigour and inclusiveness of evidence syntheses, for example, via the development of the RepOrting Standards for Systematic Evidence Syntheses [65]. Despite such advances, the ethical considerations of conducting syntheses are not often explicitly discussed [66]. Taking time and space to reflect upon how to engage with these environmental issues it itself a useful strategy. Accounting for ethical considerations is critical to ensure that the values and beliefs of different stakeholders are fairly and equitably represented [67]. Serious ethical implications can arise in a number of ways. For example, evidence syntheses are drawn upon to inform policy based on the assumption that their findings are accurate representations of a larger population (e.g., of people, organisms, policies)—but the results are drawn on biased (or non-representative) primary research—and thus are not a true representation of all stakeholders. Moreover, individual worldviews and values of researchers leading syntheses have the potential to introduce biases to the search strategy. Such biases could include, for example, funding bias, methodological bias, outcomes bias and confirmatory bias, and could lead to misrepresentation of the literature whereby a researcher steers (be it consciously or subconsciously) an outcome towards a preconceived notion. In a final example, ‘database bias’, whereby certain types of studies are more likely to be retrieved through common search strategies [68], lead to the over-representation of certain languages or geographical locations, once again leading to misrepresentation of stakeholders [69]. To ensure that syntheses are conducted in an ethically responsible manner it is critical that researchers engage explicitly and transparently with a variety of ethical issues such as those associated with issues of voice and representation [70]. Suri [67] provides a meaningful starting point for engaging with these issues, however, further work is needed to better account for ethical issues directly associated with undertaking environmental evidence syntheses.

## Opportunities for better understanding environmental evidence in action

In summary, there are many opportunities for scholarship related to evidence curation, synthesis and application. This article is intended to encourage such work and provides ideas that we deem important and profitable. As we continue to develop and implement the evidence-based environmental management field and paradigm shift, it is insufficient to simply generate evidence syntheses without understanding their use within the broader environmental management ecosystem. Key here is understanding the merits of different evidence synthesis methods, understanding the needs of decision makers, and determining how to best connect evidence with action. This will require diverse expertise beyond the “usual” experts. For example, disciplines such as social science, behavioural sciences and public policy are sorely needed to bring theoretical underpinnings and rigour to the study of evidence use (or ignorance).

These aforementioned activities are consistent with the spirit of the journal Environmental Evidence and it is for that reason that we have created a new type of article (called Evidence in Action articles) with the hopes that authors will submit their work in this space. As per the guide to authors “*Evidence in Action articles typically involve a discussion of the impact of evidence-based practice on environmental managers, of evidence synthesis on policy making, or a discussion of developments at the science-policy interface. This could be regional, national or global and may resemble a case study or more of a perspective article. Evidence in Action articles should include logical subheadings that guide the reader through the narrative*.” Consistent with the notion that there is need to better understand the evidence synthesis and use community, we encourage papers that include diverse contributors including end users, Indigenous knowledge holders, and others that are often excluded from papers. Beyond empirical research, we also welcome reflective essays that provide candid summaries based on lived experiences in the environmental evidence sphere. Beyond the new article type, more broadly we need this work and expect that this paper could yield research that is published in a variety of outlets. We envision a not-too-distant future where the enhanced understanding of the environmental evidence and evidence-based decision-making ecosystem related to the ideas presented here represents meaningful advances in our ability to realize the promise of evidence-based environmental management.

## Data Availability

Not applicable—this is a perspective article.
